# “Acing” the Hidden Spade: Review of Diagnosis, Follow-up, Prognosis, and Various Associations of Apical Variant Hypertrophic Cardiomyopathy

**DOI:** 10.7759/cureus.3979

**Published:** 2019-01-29

**Authors:** Hiren Patel, Nway L Ko Ko, Sundeep Kumar, Bernard Gros

**Affiliations:** 1 Internal Medicine, University of Central Florida College of Medicine, Orlando, USA

**Keywords:** hypertrophic cardiomyopathy, apical variant, apical septal hypertrophic cardiomyopathy, diagnosis, sudden cardiac death, ace of spade

## Abstract

Apical variant hypertrophic cardiomyopathy (AHCM) is a known entity since its first introduction by Sakamoto and Yamaguchi. However, unlike classical hypertrophic cardiomyopathy (HCM), it is less explored in terms of its associated diagnosis and long-term outcomes. Through this case presentation, we aim to have an in-depth review to help physicians identify and better understand several aspects of AHCM. Given the increased availability and utilization of high precision cardiac imaging modalities, apical septal hypertrophic cardiomyopathy will increasingly be recognized as a distinct, clinically significant variant of classical HCM. Contrast echocardiogram is the most effective and diagnostic study when performed in the right setting with high suspicion on clinical examination findings and typical electrocardiogram (EKG) findings. Cardiac magnetic resonance imaging (MRI) has equal diagnostic yield as a contrast echocardiogram. It is associated with a wide spectrum of presentation ranging from asymptomatic course with incidental findings on imaging to rarely being associated with ventricular arrhythmia. The question of utility of implantable defibrillators in individuals, particularly without any underlying arrhythmias, remains unexplained and needs further evidence-based guidance.

## Introduction

Sakamoto [1] and Yamaguchi [2] have made great contributions to the current understanding of apical variant hypertrophic cardiomyopathy (AHCM). Their studies have highlighted the electrocardiogram (EKG) and echocardiographic findings in AHCM predominantly in patients with previously unexplained marked T-wave changes on EKG. Sakamoto was able to identify that the depth of the T-wave inversion correlated with the degree of apical thickness in comparison to the mid left ventricle [1]. Unfortunately, with conventional measurements of the left ventricular walls limited to the basal segments of the septum and posterior wall, this variant of hypertrophy can be often overlooked on standard echocardiograms. As a result this entity remains a diagnostic challenge. Unlike classical hypertrophic cardiomyopathy (HCM), it is less explored in terms of its associated diagnosis and long-term outcomes. Through this case presentation, we aim to have an in-depth review to help physicians identify and better understand several aspects of AHCM.

## Case presentation

**Figure 1:**
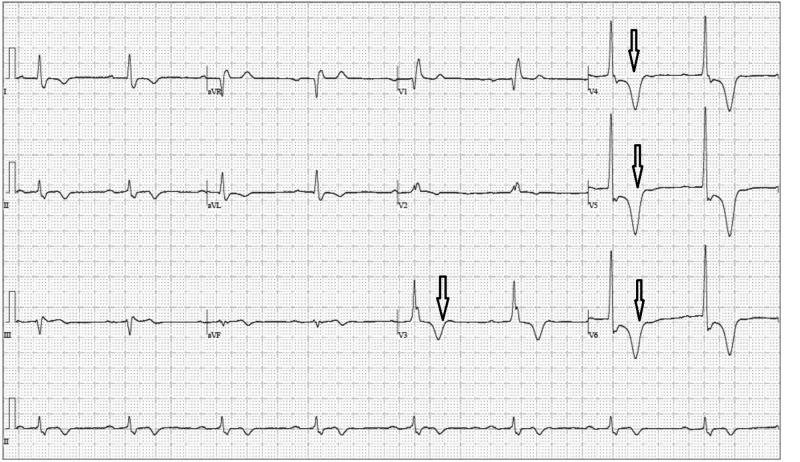
Initial electrocardiogram (EKG). Sinus bradycardia with first degree A-V block, right bundle branch block, deep T-waves in lead V3-V6 (shown by arrows).

An 83-year-old African American asymptomatic male with controlled hypertension was found to have abnormal findings upon routine EKG (sinus bradycardia with first degree A-V block, right bundle branch block, deep T-waves V3-V6) (Figure 1) done in his primary care physician’s office. Physical exam was nonsignificant, except for grade 2/6 systolic ejection murmur at left lower sternal border.

**Figure 2:**
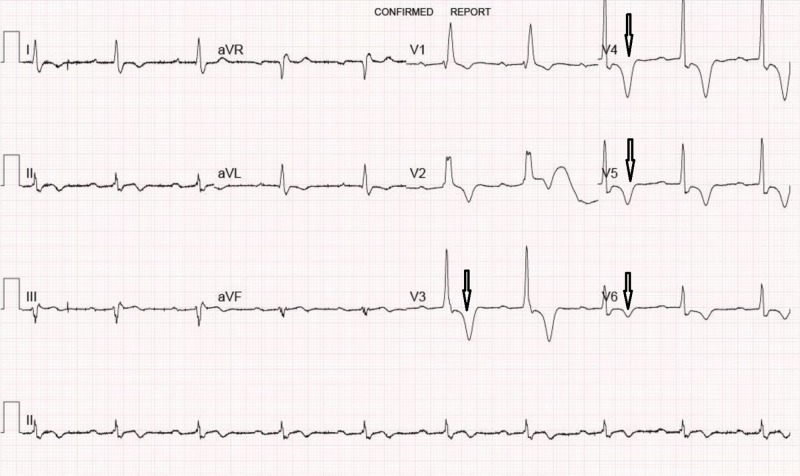
Follow-up EKG. Similar deep T-waves in leads V3-V6 as initial EKG (shown by arrows).

A transthoracic echocardiogram (TTE) showed borderline left ventricular hypertrophy (LVH) (interventricular septal dimension 11 mm and left ventricular posterior wall dimension 11 mm) with normal left ventricular systolic function (60%-65%) and minimal aortic stenosis (AVmax velocity 1.75 m/s). No further cardiac workup was pursued. A year later, the patient underwent a chest computed tomography (CT scan) for an unrelated issue. On this study, the heart was reported as “normal in size.” Three years later, his primary care provider performed another EKG and ordered another echocardiogram. A follow-up EKG (Figure 2) and echocardiogram were essentially unchanged (interventricular septal dimension 10 mm and left ventricular posterior wall dimension 10 mm) (Video 1). A cardiology consultation was requested for further evaluation of these persistent findings.

**Video 1:** Transthoracic echocardiogram (four-chamber apical view without contrast).

After initial cardiac consultation, the patient underwent nuclear treadmill stress test. Nuclear images showed prominent apical tracer uptake (at rest and peak stress) suspecting apical hypertrophy without ischemic findings. Prior echocardiograms were then reviewed and evidence for apical hypertrophy was seen that was not previously appreciated. Findings were later confirmed on echocardiogram with contrast revealing clearer evidence for apical hypertrophy and diagnostic “ace of spades” sign (Video 2). The patient denied family history of any cardiomyopathy, arrhythmias, or sudden cardiac death (SCD). No further medical management was needed, as the patient was asymptomatic. However, he was advised to undergo genetic screening for hereditary cardiomyopathy variants. He pursued comprehensive cardiomyopathy gene testing panel which showed heterozygosity for a (Leu425Pro) L425P variant of uncertain significance in the *ACTN2* gene [(CTG>CCG):c. 1274 T>C in exon 12 of the *ACTN* gene]. No deletion or duplication involving any of the nuclear genes was found. The ideal management strategy for this variant is unclear due to its rarity and paucity of published literature. With the initial work-up complete, we made two recommendations to the patient. First, he should have continued clinical follow-up. Second, his first-degree relatives should be encouraged to seek targeted genetic testing of the *ACTN2* gene.

**Video 2:** Transthoracic echocardiogram with contrast (four-chamber apical view). "Ace of spades" sign and apical systolic obliteration of left ventricle due to apical variant hypertrophic cardiomyopathy.

## Discussion

Incidence and prevalence

While hypertrophic cardiomyopathy (HCM) is the most common cause of cardiac arrest in individuals younger than 40 years of age, its apical variant has a distinctly different epidemiological prevalence and presentation. The prevalence of HCM in the general population is approximately 1 out of every 500 persons; however, the apical variant is less common. In a prior 2014 case report, Ho et al. highlighted that AHCM represents a small minority of the known cases of HCM (prevalence of HCM 0.2%-0.5%), constituting 15%-25% of cases in Chinese and Japanese cohorts and 3% of cases in American cohorts [3]. In Japan, AHCM appears to represent distinctive variation from classical HCM (in Japan or elsewhere) in which genetic transmission is known and marked symptoms are relatively common [4]. The basis for differences in the phenotypic expression of apical hypertrophy between Asians and non-Asians has not been elucidated [5]. 

Symptoms and presentation

It is mostly asymptomatic and can be detected incidentally in EKG like in this case. Pathognomonic EKG findings are high QRS voltage, repolarization abnormalities and deep T-wave inversions (giant negative T-waves) [1] (defined as a voltage of negative T-wave greater or equal to 1 mV), and a high R-wave voltage in septal leads (especially V3) [6]. The clinical presentation of apical HCM can sometimes be mistaken for an acute coronary syndrome when patients present with chest pain and repolarization abnormalities on the EKG [6]. In these cases, coronary angiography would lack findings of coronary artery disease and can depict classical ventriculographic “spade like” configuration of left ventricular cavity.

Diagnosis

So far, the diagnostic studies have been TTE with contrast, cardiac magnetic resonance imaging (CMR) and nuclear stress test. Given the initial asymptomatic presentation of most of the AHCM and poor echocardiographic visualization of the apical segment [6], noncontrast TTE studies have been shown to be seldom diagnostic. The depth/magnitude of the T-wave inversions in lateral leads is not solely established to be associated with the diagnosis. However, concurrent findings of a systolic ejection murmur and/or family history of SCD/HCM should raise suspicion for AHCM. Prompt evaluation with contrast TTE should be undertaken in these patients. At this stage, there has not been any pathognomonic EKG or physical exam findings attributed to it, hence making the diagnosis a true challenge in asymptomatic patients. TTE without contrast is of limited utility for diagnosis; however, a study with contrast shows diagnostic findings while CMR shows localized hypertrophy of apical septum with spade sign at end-diastole and obliteration of apical ventricular cavity at end systole [7].

Repolarization abnormalities with LVH are also seen in hypertensive heart disease, a significantly more prevalent pattern of disease when compared to AHCM. However, the EKG findings in our patient were clearly discordant with his known well-controlled hypertension. In addition, his initial echocardiogram did not show significant hypertrophy of the septum and posterior wall (values being in upper limit of normal for wall thickness). He was misdiagnosed as undertreated hypertensive heart disease; however, aggressive treatment with antihypertensive agents did not curtail this manifestation. Also, hypertensive heart disease manifests more commonly as concentric LVH in contrast to this patient’s left ventricular structural presentation.

Treatment

The known risk factors for SCD in classical HCM have been categorized in a consensus document from the American College of Cardiology and the European Society of Cardiology as “major” and “possible” risk factors in individual patients [8]. [Major risk factors include prior cardiac arrest, spontaneous sustained ventricular tachycardia (VT), spontaneous nonsustained VT, family history of SCD, unexplained syncope, left ventricular thickness greater than or equal to 30 mm, and an abnormal blood pressure response to exercise]. However, AHCM lacks such evidence and guidelines due to low incidence and lack of evidence. In some instances, HCM risk—SCD score has been used to determine candidates for implantable cardiac defibrillator (ICD) placement [6], but this practice lacks general validation and needs further insight. Literature analysis shows two patients with symptomatic presentations of AHCM who underwent ICD placement. Neither patient had firing of their ICD within six months of placement [5]. Investigative studies to assess the need for ICD placement would be anticipated to include family history of HCM, SCD, and/or personal history of VT. Given lack of supporting family history, concerning genetic study results or most importantly, any active symptoms from the underlying hypertrophic variant, a shared decision was made with our patient to not pursue provocative electrophysiological study and/or implantation of ICD.

Complications and outcomes

As shown by a retrospective study from Toronto General Hospital, it is believed that AHCM carries a benign prognosis in terms of cardiovascular mortality. For those who do experience serious cardiovascular complications, most common ones are myocardial infarction and arrhythmias (atrial fibrillation) [9]. It appears to be less benign in Western countries than in Japan, with atrial fibrillation and myocardial infarction the most frequent complication occurring in up to a third of patients during long-term follow up, rather than sudden death [10]. Lee and colleagues in their report on clinical impact of atrial fibrillation (AF) in patients with AHCM (n = 306), showed that AF occurred in 25.2% of these patients [11-12]. Although generally associated with a better prognosis than other forms of HCM, serious cardiac complications have been described, including progressive heart failure, myocardial infarction, and SCD [9, 13]. Female gender and atrial fibrillation have been identified as predictors of mortality in retrospective studies [14]. Despite the higher prevalence of this condition among Japanese patients, clinical presentation and long-term cardiovascular outcomes appear to be similar [15]. Specifically, long-term follow up studies have shown co-morbid atrial fibrillation, apical myocardial infarction, ventricular arrhythmia, and apical thrombosis with subsequent embolization to occur in up to 33% of all patients irrespective of ethnicity [16]. Prognosis of associated aneurysm with AHCM is unknown. However, HCM with apical aneurysms is associated with considerable morbidity and mortality [17].

Genetics, follow-up, and secondary prevention

A very limited number of sarcomere gene defects, and in particular, cardiac actin (ACTN) Glu101Lys, have been shown to consistently produce the AHCM phenotype [18]. Among non-Asian population, some case studies have shown that there has been an anticipation of higher genetic predisposition amongst young African American patients [5, 19], while other studies do not have such congruent findings and describe predisposition in Caucasian patients. The long-term outcomes for AHCM are unclear. It is imperative to establish a genetic database or patient registry to assimilate the genetic variations associated with this condition, so that patients found to have these mutations can be properly counseled regarding their long-term prognosis.

No definitive guidelines delineate the role for defibrillator implantation in AHCM patients with family history of SCD. Not surprisingly, expert consensus supports the use of implantable defibrillator for primary prevention in select high risk patients, notably those with one of the five following risk factors: a family history of SCD, unexplained syncope, asymptomatic nonsustained VT, an abnormal blood pressure response to exercise, and a left ventricular wall thickness > 30 mm [20].

Important questions to be answered

1. Do apical and nonapical variant carry equal genetic inheritance and familial incidence?

2. Is there a need for familial genetic screening indicated for every patient with apical variant HCM?

3. Is there a genetic variation in presentation and occurrence of apical HCM in Asian vs. non-Asian populations?

4. Is there a genetic mutation similar to that caused by mutations in one of nine genes encoding sarcomeric proteins as in traditional HCM?

5. How often do we follow-up with patient with AHCM, symptomatic vs. asymptomatic?

6. Can pathognomonic EKG findings be enough grounds to perform a contrast echocardiogram as an initial imaging study for definitive diagnosis?

7. Do asymptomatic patients need ICD?

## Conclusions

Given the increased availability and utilization of high precision cardiac imaging modalities, apical septal hypertrophic cardiomyopathy will increasingly be recognized as a distinct, clinically significant variant of classical HCM. Contrast echocardiogram is the most effective and diagnostic study when performed in the right setting with high suspicion on clinical examination findings and typical EKG findings. CMR has equal diagnostic yield as a contrast echocardiogram. It is associated with wide spectrum of presentation ranging from asymptomatic course with incidental findings on imaging to rarely being associated with ventricular arrhythmia. The question of utility of implantable defibrillators in individuals, particularly without any underlying arrhythmias, remains unexplained.
